# Effects of periarticular injection on analgesic effects and NSAID use in total knee arthroplasty and total hip arthroplasty

**DOI:** 10.6061/clinics/2017(12)03

**Published:** 2017-12

**Authors:** Wen-rui Ban, Ery-ang Zhang, Lei-feng Lv, Xiao-qian Dang, Chen Zhang

**Affiliations:** The First Department of Orthopedics, the Second Affiliated Hospital of Medical College, Xi’an Jiaotong University, Xi’an Shaanxi, 710004, P. R China

**Keywords:** Arthroplasty, Pain, Knee, Hip, Analgesia, Nonsteroidal Anti-Inflammatory Drugs

## Abstract

**OBJECTIVES:**

This study examined periarticular multimodal drug injection and the use of nonsteroidal anti-inflammatory drugs for an early analgesic effect after total knee arthroplasty and total hip arthroplasty. Patient satisfaction and benefits from the treatment were also assessed.

**METHODS:**

A total of 110 patients who were scheduled to undergo total knee arthroplasty and 86 patients who were scheduled to undergo total hip arthroplasty were divided into two groups, the study group and the control group. The study group received a periarticular multimodal drug injection during surgery. The control group received an equal volume of normal saline. All patients received an analgesia pump and a moderate dose of nonsteroidal anti-inflammatory drugs. Resting and motion Numeric Rating Scale scores, the Western Ontario and McMaster Universities Arthritis Index, knee or hip joint range of motion, length of postoperative hospital stay, patient satisfaction, total nonsteroidal anti-inflammatory drug consumption and side effects were recorded.

**RESULTS:**

Both study groups exhibited significant improvement in pain Numeric Rating Scale scores during rest and exercise several days after the surgery. The range of joint motion was greater in the study group, and the length of postoperative hospital stay was shorter than that in the control group. Patients in the study group consumed fewer nonsteroidal anti-inflammatory drugs and reported greater satisfaction with surgery.

**CONCLUSION:**

Intraoperative periarticular multimodal drug injection significantly relieved pain after surgery and reduced nonsteroidal anti-inflammatory drug consumption. These patient had a better postoperative experience, including satisfaction and rehabilitation.

## INTRODUCTION

Total knee arthroplasty (TKA) and total hip arthroplasty (THA) are important treatments for severe joint disease that can relieve joint pain and improve joint function. Approximately 90% of patients with pain and dysfunction show improvement after TKA, which also improves their quality of life. Most patients (85%) are very satisfied with the curative effect of surgery and postoperative quality of life 1,2. However, postoperative pain has a harmful effect on important organs and directly affects postoperative rehabilitation exercises 3,4. Pain after surgery increases patients’ concerns about rehabilitation exercises, which affects the rehabilitation process. Moreover, the clinical application of many analgesic methods is limited 5-7. Therefore, identification of effective and safe analgesic treatments is an important challenge. Periarticular injection effectively prevents pain and leads to a lower systemic side effect profile. This randomized, single-blind study aimed to compare the analgesic effects of periarticular injection in TKA and THA postoperative patients and nonsteroidal anti-inflammatory drug (NSAID) use.

## MATERIALS AND METHODS

### Patient population

Approval was obtained from the Biomedical Ethics group of the medical department of Xi’an Jiaotong University (number 2012-693). From 2012.9.1 - 2015.11.01, we conducted a prospective, randomized single-blinded study of all eligible men and nonpregnant women who were scheduled to undergo primary TKA and THA for osteoarthritis. All patients were at least 20 years old and no more than 80 years old. The following inclusion criteria were used: (1) the American Society of Anesthesiologists (ASA) grade I - III rating; (2) weight index of 20 - 35 kg/m^2^; (3) unilateral TKA and THA; (4) ability to cooperate with the investigation and understand the pain score; and (5) provision of informed consent.

**Figure 1:**
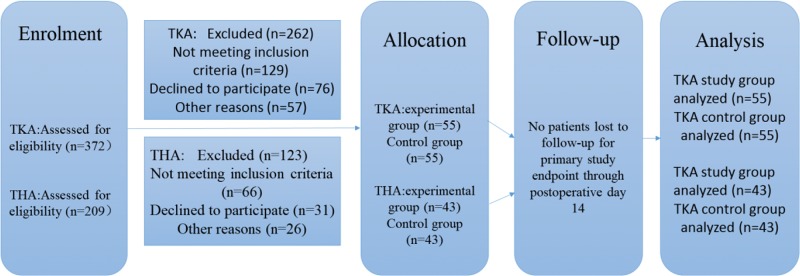
Subject inclusion decision tree.

The following exclusion criteria were applied: (1) did not meet the above requirements; (2) nervous or mental disorders; (3) known drug allergy or tolerance to study drugs; (4) renal insufficiency or abnormal liver enzymes; (5) acute or chronic knee infection; and (6) serious cardiovascular disease, diabetes, or uncontrolled angina. All patients were randomly divided into two groups. No significant differences in baseline demographics were observed between the two groups (Table 1). Figure 1 shows a detailed flowchart describing our selection criteria and decision-making process in determining study participants.

### Patient grouping

A biostatistician who was blinded to the research study generated a random number sequence with a simple random number table. All random number cards were settled in sealed envelopes. Patients were allocated either to the study group or control group based on whether the random number was odd or even when he or she was enrolled. Injections in the study group consisted of 200 mg of ropivacaine, 30 mg of ketorolac, 5 mg of hexadecadrol, and 0.3 ml of epinephrine. These drugs were mixed in a sterile normal saline solution with a combined volume of 50 ml. The control group received an equal volume of normal saline solution.

All patients received general anesthesia, and the same surgeon performed all surgeries. All patient implants are from Johnson & Johnson of America. The mixed drugs were injected in the entire length of the wound, especially in the subcapsular muscles and subcuticular tissues. All patients received patient-controlled analgesia (2 µg/kg sufentanil and 0.2 mg/kg tropisetron mixed with sterile normal saline solution in a total volume of 100 ml), and we limited the drip rate to 2 ml/h. Patients were administered moderate NSAIDs (celecoxib) based on their needs 6 h postoperatively.

All patients were asked to perform ankle pump exercises during the 24 h after surgery and active flexion movement 24 h after surgery. Pain scores, Western Ontario and McMaster Universities Arthritis Index (WOMAC) scores, adverse reactions, range of motion and use of NSAIDs were recorded daily. The average of the measurements was calculated. Patient satisfaction was assessed on the seventh day after surgery.

### Observation indexes

Pain scores were measured using the Numeric Rating Scale score (NRS), where 0 indicated no pain and 10 indicated the worst imaginable pain. We recorded the pain score, range of motion, and NSAID use 12 h, 24 h, 48 h, 72 h, 4 d, 5 d, 6 d, 7 d, and 14 d after surgery. The number of reports of nausea, vomiting and other adverse reactions was recorded during hospitalization. Evaluations of satisfaction, including pain control, hospitalization days, functional recovery and general satisfaction, were recorded on the seventh day after surgery.

### Statistical analysis

Statistical interactions between the two groups and time were assessed to determine the longitudinal effects of group on the outcome of interest. Enumeration data were evaluated using the chi-square test, and measurement data were analyzed using t tests. Our sample size estimate was based on the WOMAC score. According to the results of our pre-experiment (n=10), we defined an important between-group difference to be approximately 2 points in the TKA group and 3 point in THA group 2 to 4 days post-arthroplasty. The power was set at 0.80 and the overall type I error probability at 0.05. Applying these assumptions yielded an approximate required sample size of 41 subjects in the TKA groups and 36 subjects in the THA groups. Because many of the late eligible patients volunteered to join, we expanded the sample size.

All information was processed using SPSS 23.0. Statistical significance was set at *p*<0.05.

## RESULTS

**Figure 2:**
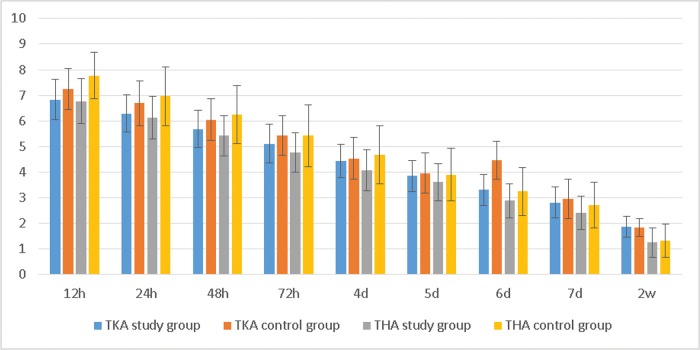
The NRS scores with activity. *The average NRS score with activity in the TKA study group was lower than that in the control group at 12 h, 24 h, 48 h, and 72 h; the average NRS score in the THA group was lower than that in the control group at 12 h, 24 h, 48 h, 72 h, 4 d and 6 d.

We found that NRS scores with activity within a few days after surgery were lower in the study group than in the control group (Table 2, Figure 2). Patients in the THA study group also reported less pain than those in the TKA study group.

**Figure 3:**
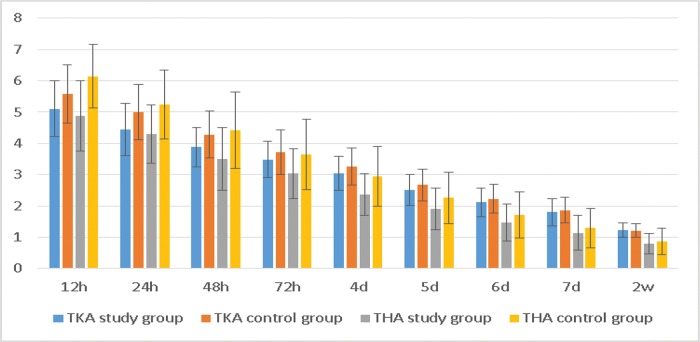
*The NRS scores at rest. The average score in the TKA study group was lower than that in the control group at 12 h, 24 h, and 48 h, whereas the average score in the THA study group was lower than that in the control group at 12 h, 24 h, 48 h, 72 h, 4 d and 5 d.

The NRS scores at rest also displayed the same trend (Table 3, Figure 3).

**Figure 4:**
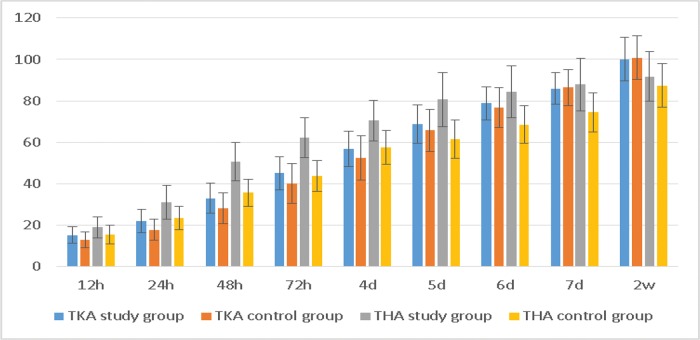
Range of motion.

The TKA study group showed a larger range of knee flexion within 4 days postoperatively than the control group, whereas the THA study group showed a larger range of hip flexion within 7 days postoperatively (Table 4, Figure 4). These differences were significant (*p*<0.05).

**Figure 5:**
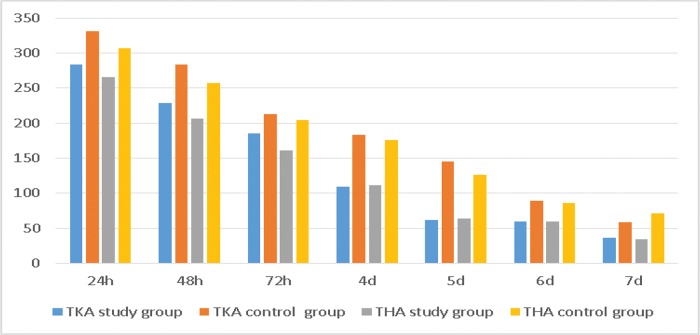
NSAID consumption.

The study group was administered a dose of celecoxib that was far lower than that in the control group. The TKA study group received a total of 965 mg of Celebrex, while the control group received 1,325 mg in one week (*p*<0.05). The THA study group was treated with 918 mg of Celebrex, while the control group was treated with 1,238 mg in one week (*p*<0.05) (Table 5, Figure 5).

**Figure 6:**
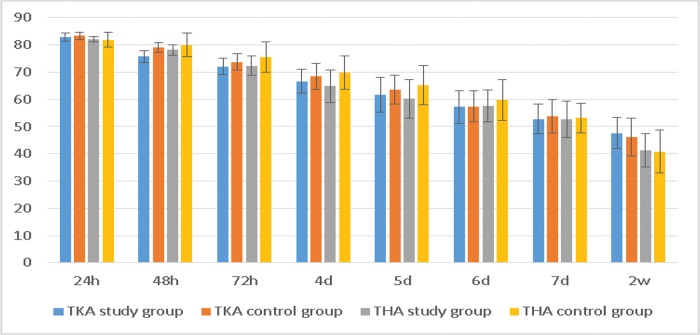
WOMAC scores after the operation.

The WOMAC scores of the TKA study group were significantly better than those of the control group at 48 h, 72 h, and 4 d, and the scores of the THA study group were better than those of the control group at 48 h, 72 h, 4 d and 5 d (Table 6, Figure 6). These differences were significant (*p*<0.05).

The length of postoperative hospital stay was significantly different between groups. The hospital stay duration in the TKA study group was 9.25±1.99 days, compared to 10.44±2.62 days in the control group (*p*<0.05), whereas the hospital stay duration in the THA study group was 8.08±2.11 days, compared to 10.06±2.59 days in the control group (*p*<0.05).

We found that postoperative pain satisfaction was significantly higher in both study groups than in the control group. We also observed that functional recovery and general satisfaction in the THA study group were significantly higher than in the control group (*p*<0.05). However, no significant differences in the degree of satisfaction related to hospitalization days were observed.

## DISCUSSION

Adequate postoperative pain control in total joint replacement (TJR) during postoperative functional recovery is very important, and a sufficient analgesic effect can help relieve pain, allow patients to get out of bed earlier to exercise, restore joint function, and prevent the formation of deep vein thrombosis (DVT) 8. Severe pain also leads to prolonged hospital stays and increased opioid use. Many treatment options for joint replacement postoperative analgesia exist, but all these options have limitations. For instance, epidural analgesia frequently causes complications, such as nausea, hypotension, and urinary retention. TJR patients often receive anticoagulant drugs to prevent DVT, but these agents increase the risk of operative bleeding 9,10. The effect of intravenous analgesia is poor, and patients are prone to respiratory depression, nausea, vomiting, itching, and sleepiness 10,11. We compared resting and motion NRS scores, joint range of motion, WOMAC scores, time of active exercise, length of postoperative hospital stay, patient satisfaction, total NSAID consumption and side effects between the two groups to identify safer and more effective perioperative analgesic methods. Our results demonstrated that periarticular injection for postoperative pain control led to good patient recovery and effectively reduced the use of NSAIDs, which can cause serious side effects. The limitations of our study include the short-term follow-up period and that patients may not have effectively evaluated their situation and NSAID drug use.

Our study found that NRS scores with activity or rest were lower in the study group than in the control group during the early perioperative period, and previous studies have reported similar findings 11-15. Busch et al. 11 found that the combination of ropivacaine and ketorolac tromethamine as a periarticular multimodal drug injection effectively reduced the intake of opioids, and postoperative NRS scores at 6 h, 12 h, and 24 h were significantly lower than those in the control group during the perioperative period of TKA. Wei Liu et al. 12 found that the average NRS score of THA study patients was significantly lower than that in the control group. Todd et al. 13 performed a randomized double-blind controlled study and found that the combination of ropivacaine, adrenaline and ketorolac tromethamine exhibited a better effect than the use of ropivacaine and epinephrine alone, and it effectively reduced the amount of morphine for analgesia 16. Dexamethasone as a long-term glucocorticoid exhibits strong anti-inflammatory effects, and ropivacaine or bupivacaine combined with dexamethasone prolongs the effect of local anesthetics 17. Therefore, different drugs and drug delivery methods exhibit different effects, and these differences require further research. In addition, on postoperative days 2 to 3, the experimental group showed a better analgesic effect, and this difference gradually disappeared after 3 days. Although there was still a statistically significant difference between the two groups, the difference is very subtle and soon disappeared. This is because the periarticular multimodal drug injection effect is relatively short. We also noted that the effect of TKA postoperative analgesia between the two groups is subtle, which indicates that intraoperative periarticular multimodal drug injection may not be sufficient to completely control postoperative pain in TKA patients. All patients reported mild pain after 1 week.

Patients with better pain relief exhibited improved postoperative motion and WOMAC scores. This study demonstrated that the range of keen flexion of the TKA study group patients within 4 days and that of the THA study group patients within 6 days after surgery were better than that in the control group. Early postoperative exercise is beneficial for postoperative rehabilitation and may prevent some treatment complications 16-20. In fact, similar to the VAS score trend, this difference is not obvious in TKA. In particular, the difference in WOMAC scores between the two groups is subtle because the WOMAC score is divided into three parts; pain is only one part of it, and there were no significant differences between the two groups in many items of the WOMAC score.

We focused on the use of NSAIDs after surgery. Celecoxib capsules are a relatively new selective COX-2 inhibitor that primarily inhibit a COX-2 zymoprotein to block the synthesis of prostaglandins and achieve anti-inflammatory effects. Numerous studies have demonstrated that patients who receive celecoxib exhibit a reduced incidence of nausea and vomiting (28%). However, no research has used large samples to assess the side effects of celecoxib 23. Indeed, the side effects of postoperative celecoxib analgesia remain a problem that is difficult to ignore 23,24. Our results demonstrated that the study group ingested much less celecoxib than the control group. Six patients in our study reported nausea and vomiting in the TKA study group compared to 7 patients in the TKA control group, while 8 patients in the THA group reported side effects. No cardiac or central nervous system toxicity was observed. No significant differences were observed between the two groups, although the long-term side effects of drug treatment require further study. Indeed, previous studies have suggested that the long-term use of NSAIDs significantly increases the incidence of adverse reactions 25. Our results showed that periarticular multimodal drug injection significantly reduced the intake of NSAIDs, which may reduce the discomfort caused by NSAIDs. In this study, we recommended that patients take no more than 200 mg daily, unless there is a special need. Therefore, the difference between the two groups was not very large but was sufficient to show that periarticular multimodal drug injection can effectively reduce the intake of NSAIDs. With the development of new technologies, the use of NSAIDs has shown a decreasing trend, but many drug guides still recommend oral NSAIDs, which is why we selected NSAIDs to study. The ultimate goal is elimination of NSAID for pain control in the future

We applied a digital classification method for the patients to evaluate all aspects of satisfaction during hospitalization. We found that postoperative pain satisfaction in the study group was significantly higher than that in the control group, and we also found that functional recovery and general satisfaction in the THA study group were significantly higher than in the control group. However, no significant differences existed in the degree of satisfaction with the number of hospitalization days.

THA patients showed better pain control and rehabilitation satisfaction than TKA patients. This result may be because TKA patients feel more pain after surgery than THA patients; thus, periarticular injection may be effective in controlling postoperative pain in THA patients but may not be sufficient to completely control postoperative pain in TKA patients. Additionally, this difference between groups may also be related to operation time, bleeding volume, degree of tissue damage, patient expectations, and other relevant issues, although these factors were not analyzed in the current study. Our results suggest that we cannot focus only on pain control after surgery in TJR patients; instead, a variety of measures should be used to improve the prognosis of patients and their comprehensive quality of life.

Periarticular multimodal drug injection is a simple, safe surgery that effectively reduced the pain of patients after TKA and THA, and this method effectively advanced the time that patients began joint exercises, improved joint function recovery, and shortened hospital stay. Local dosing in the joint reduced the intake of NSAIDs, which may reduce NSAID-induced damage to the digestive tract and kidney. We believe that with the development of new technologies, the use NSAID for pain control after TKA and THA will decrease.

## AUTHOR CONTRIBUTIONS

Ban WR made substantial contributions to the conception and design of the manuscript, acquisition of the data, analysis and interpretation of the data and manuscript drafting. Lv LF collected the range of motion and the patient satisfaction data. Zhang EA collected the NRS and WOMAC scores. Dang XQ and Zhang C conceived the study and participated in its design and coordination, revised the manuscript critically for important intellectual content and approved the final version of the manuscript. All authors read and approved the final version of the manuscript.

## Figures and Tables

**Table 1:** Demographic data for patients in the study.

	TKA study group (n=55)	TKA control group (n=55)	THA study group (n=43)	THA control group (n=43)
**Age**	63±11	65±9	58±11	55±13
**Sex (male: female)**	13:42	15:40	19:24	18:25
**BMI**	24.16±2.69	24.09±3.11	23.35±2.23	22.90±3.56
**HSS score**	49.45±12.96	49.02±14.23		
**Harris score**			46.87±13.29	43.42±15.27
**WOMAC score**	49.10±13.18	50.63±14.02	41.30±12.73	38.51±14.92

All data except sex are denoted as the mean±SD. There was no significant difference between the two groups.

WOMAC score: Western Ontario and McMaster Universities Arthritis Index.

**Table 2:** The NRS scores with activity.

	TKA study group	TKA control group	t-value	*p*-value	THA study group	THA control group	t-value	*p*-value
**12 h**	6.83±0.78	7.25±0.80	-2.832	0.006	6.77±0.88	7.77±0.90	-5.185	<0.001
**24 h**	6.29±0.73	6.69±0.88	-2.567	0.012	6.13±0.82	6.96±1.14	-3.894	<0.001
**48 h**	5.68±0.72	6.05±0.82	-2.483	0.015	5.42±0.79	6.25±1.13	-2.980	0.004
**72 h**	5.11±0.75	5.43±0.77	-2.191	0.031	4.77±0.78	5.42±1.21	-2.071	0.041
**4 d**	4.43±0.65	4.54±0.82	-0.757	0.451	4.07±0.80	4.68±1.13	-2.182	0.032
**5 d**	3.85±0.60	3.96±0.79	-0.839	0.403	3.61±0.72	3.90±1.03	-1.495	0.139
**6 d**	3.31±0.60	4.47±0.74	-1.219	0.225	2.89±0.66	3.25±0.93	-2.067	0.042
**7 d**	2.82±0.60	2.96±0.76	-1.092	0.277	2.40±0.65	2.72±0.90	-1.933	0.057
**14 d**	1.86±0.41	1.84±0.36	0.245	0.807	1.26±0.57	1.33±0.65	.0.562	0.576

**Table 3:** The NRS scores at rest.

	TKA study group	TKA control group	t-value	*p*-value	THA study group	THA control group	t-value	*p*-value
**12 h**	5.10±0.89	5.58±0.94	-2.788	0.006	4.88±1.13	6.14±1.01	-5.444	<0.001
**24 h**	4.44±0.84	5.00±0.88	-3.423	0.001	4.29±0.93	5.24±1.10	-4.301	<0.001
**48 h**	3.88±0.63	4.28±0.75	-3.018	0.003	3.50±1.01	4.41±1.22	-3.750	<0.001
**72 h**	3.48±0.58	3.72±0.71	-1.965	0.052	3.03±0.79	3.65±1.12	-2.996	0.004
**4 d**	3.05±0.54	3.26±0.60	-1.893	0.061	2.37±0.67	2.94±0.96	-3.247	0.002
**5 d**	2.51±0.49	2.67±0.50	-1.747	0.084	1.90±0.66	2.26±0.83	-2.213	0.030
**6 d**	2.12±0.46	2.23±0.46	-1.240	0.218	1.48±0.59	1.72±0.74	-1.674	0.098
**7 d**	1.80±0.43	1.86±0.41	-0.797	0.427	1.14±0.56	1.30±0.63	-1.227	0.223
**14 d**	1.24±0.23	1.21±0.22	0.549	0.584	0.79±0.33	0.86±0.42	-0.910	0.366

**Table 4:** Range of motion after the operation.

	TKA study group (°)	TKA control group (°)	t-value	*p*-value	THA study group (°)	THA control group (°)	t-value	*p*-value
**12 h**	15.33±3.92	13.04±3.70	3.253	0.002	19.06±5.16	15.55±4.67	3.306	0.001
**24 h**	22.18±5.59	17.85±5.13	4.231	<0.001	31.08±8.12	23.54±5.72	4.954	<0.001
**48 h**	33.05±7.17	28.22±7.34	3.497	0.001	50.69±9.27	35.71±6.49	8.644	<0.001
**72 h**	45.16±7.87	40.05±9.64	3.045	0.003	62.23±9.62	43.84±7.29	9.957	<0.001
**4 d**	56.91±8.42	52.64±10.74	2.321	0.022	70.56±9.84	57.66±8.23	6.582	<0.001
**5 d**	68.82±9.26	65.76±10.14	1.650	0.102	80.66±12.96	61.54±9.20	7.858	<0.001
**6 d**	78.80±7.94	76.91±9.64	1.123	0.264	84.48±12.58	68.64±8.90	6.716	<0.001
**7 d**	86.00±7.72	86.53±8.70	0.336	0.737	88.01±12.68	74.52±9.47	5.570	<0.001
**14 d**	100.15±10.51	100.84±10.56	0.344	0.732	91.79±11.96	87.45±10.59	1.780	0.079

**Table 5:** NSAID consumption.

	TKA study group	TKA control group	t-value	*p*-value	THA study group	THA control group	t-value	*p*-value
**24 h**	283.64±83.36	330.91±85.79	-2.931	0.004	265.91±77.59	307.14±83.79	-2.369	0.020
**48 h**	229.09±68.51	283.64±78.80	-3.874	<0.001	206.82±92.50	257.14±76.93	-2.736	0.008
**72 h**	185.45±55.84	212.73±47.35	-2.763	0.007	161.36±57.93	204.76±43.91	-3.901	<0.001
**4 d**	109.09±84.49	183.64±60.14	-5.331	<0.001	111.36±81.32	176.19±57.63	-4.247	<0.001
**5 d**	61.82±80.49	145.45±78.92	-5.502	<0.001	63.64±80.96	126.19±85.71	-3.481	0.001
**6 d**	60.00±78.41	89.09±83.16	-1.888	0.062	59.09±75.69	85.71±84.31	-1.542	0.127
**7 d**	36.36±64.88	58.18±73.76	-1.647	0.102	34.09±47.95	71.43±63.58	-3.084	0.003

**Table 6:** WOMAC scores after the operation.

	TKA study group	TKA control group	t-value	*p*-value	THA study group	THA control group	t-value	*p*-value
**24 h**	82.88±1.39	83.29±1.48	-1.497	0.137	82.14±0.93	81.88±2.80	0.572	0.569
**48 h**	75.68±2.22	79.14±1.79	-8.997	<0.001	78.18±1.94	80.02±4.29	-2.586	0.011
**72 h**	72.05±3.01	73.69±3.04	-2.834	0.005	72.39±3.58	75.62±5.54	-3.228	0.002
**4 d**	66.69±4.29	68.48±4.73	-2.075	0.040	64.91±5.97	69.79±6.06	-3.757	<0.001
**5 d**	61.71±6.42	63.60±5.31	-1.683	0.095	60.18±7.03	65.21±7.18	-3.285	0.001
**6 d**	57.23±6.02	57.44±5.60	-0.187	0.852	57.48±5.85	59.69±7.46	-1.535	0.128
**7 d**	52.76±5.44	53.88±6.07	-1.018	0.311	52.68±6.63	53.12±5.40	-0.334	0.739
**14 d**	47.66±5.78	46.18±6.95	-1.214	0.228	41.34±6.17	40.86±7.80	0.334	0.739
